# Gene identification for risk of relapse in stage I lung adenocarcinoma patients: a combined methodology of gene expression profiling and computational gene network analysis

**DOI:** 10.18632/oncotarget.8723

**Published:** 2016-04-13

**Authors:** Vienna Ludovini, Fortunato Bianconi, Annamaria Siggillino, Danilo Piobbico, Jacopo Vannucci, Giulio Metro, Rita Chiari, Guido Bellezza, Francesco Puma, Maria Agnese Della Fazia, Giuseppe Servillo, Lucio Crinò

**Affiliations:** ^1^ Medical Oncology, S. Maria Della Misericordia Hospital, Perugia, Italy; ^2^ Department of Experimental Medicine, University of Perugia, Perugia, Italy; ^3^ Department of Surgical and Biomedical Science, University of Perugia, Perugia, Italy; ^4^ Department of Experimental Medicine, Section of Anatomic Pathology and Histology, Perugia, Italy

**Keywords:** lung adenocarcinoma, gene expression profiling, gene networks, computational biology, cancer systems biology

## Abstract

Risk assessment and treatment choice remains a challenge in early non-smallcell lung cancer (NSCLC). The aim of this study was to identify novel genes involved in the risk of early relapse (ER) compared to no relapse (NR) in resected lung adenocarcinoma (AD) patients using a combination of high throughput technology and computational analysis. We identified 18 patients (n.13 NR and n.5 ER) with stage I AD. Frozen samples of patients in ER, NR and corresponding normal lung (NL) were subjected to Microarray technology and quantitative-PCR (Q-PCR). A gene network computational analysis was performed to select predictive genes. An independent set of 79 ADs stage I samples was used to validate selected genes by Q-PCR.

From microarray analysis we selected 50 genes, using the fold change ratio of ER versus NR. They were validated both in pool and individually in patient samples (ER and NR) by Q-PCR. Fourteen increased and 25 decreased genes showed a concordance between two methods. They were used to perform a computational gene network analysis that identified 4 increased (HOXA10, CLCA2, AKR1B10, FABP3) and 6 decreased (SCGB1A1, PGC, TFF1, PSCA, SPRR1B and PRSS1) genes. Moreover, in an independent dataset of ADs samples, we showed that both high FABP3 expression and low SCGB1A1 expression was associated with a worse disease-free survival (DFS).

Our results indicate that it is possible to define, through gene expression and computational analysis, a characteristic gene profiling of patients with an increased risk of relapse that may become a tool for patient selection for adjuvant therapy.

## INTRODUCTION

Lung cancer is the most common cause of death from cancer worldwide, and non-small cell lung cancer (NSCLC: 20% squamous and 80% adenocarcinoma (AD) histology) accounts for almost 80% of such deaths [1]. Approximately 25-30% of patients with NSCLC present with localized disease at the time of diagnosis and undergo surgery with curative intent. Despite complete tumor resection, however, the 5-year survival of these patients is poor and 40 to 70% of them will develop systemic disease with or without local relapses and will eventually die from it. No reliable clinical or molecular predictors are currently available to identify those at high risk for developing recurrent disease and the tumor-node-metastasis (TNM) staging system [2] remains the most powerful tool to predict prognosis in these patients. After radical surgery, adjuvant cisplatin-based chemotherapy (four cycles) has been established as the standard care in stage II and III patients with good performance status, rapid postoperative recovery and adequate organ function based on the results of three phase III trials and of the LACE meta-analysis showing an absolute gain of 5.3% in 5-year survival [3]. The role of adjuvant chemotherapy in stage I radically resected diseaseremains mostly undetermined due to both underrepresentation in clinicaltrials [4] and the survival benefit expected to be small in this population. Most guidelines state that it can be considered for selected patients (younger age, good performance status, large tumors, visceral pleural invasion and inadequate staging) with stage IB (sixth TNM classification) disease.

Advances in genome-wide sequencing and microarray analysis have stimulated research in molecular prognostics and have allowed the identification of molecular signatures that can promote a more precise classification and prognostication of human cancers. Recentstudies in patients with early-stage NSCLC have shown that genomic profiles constructed from patient series with long-term follow-up are able to outperform standard pathologic TNM staging in estimating risk ofdisease recurrence [5–9]. However, the signatures often contain large numbers of genes with limited information about their functional importance. This problem limits the clinical application of those signatures. The aim of this study was to identify novel genes increased or decreased in early relapse (ER) compared to no relapse (NR) lung cancer AD stage I patientsand determine the role of a specific signature of the tumor to predict patient prognosis. In this study we used a computational biology approach to construct a survival-related gene network in ADs and to identify genes which were consistently co-expressed with many survival-related genes important in multiple biological processes. The goal is to provide clinical oncologists with more information on tumor biology which could guide therapeutic interventions.

## RESULTS

### Patient characteristics

We identified 18 patients with stage I disease (TNM version 7.0) from a series of 110 consecutive early stage radically resected AD patients who were referred to the Thoracic Surgery Unit of the Perugia University Hospital, Italy. Thirteen of these 18 patients were defined as NR (patients without evidence of relapse: median follow-up 133.7 months, range 51.6-145.8) while 5 of 18 were classified as ER (patients relapsed within a year: median follow-up 10.4 months, range 0.5-54.2). Normal lung (NL) tissue taken from distant or contralateral lung from patients with AD was used as calibrator for microarray and quantitative PCR (Q-PCR) analysis. Clinical and pathologic characteristics of the two groups (n.13 NR and n.5 ER) are shown in Table 1. Median age was 64 (range 44-84) in NR patients and 67.4 (range 56-77) in ER patients; most of them were male (76.9% NR, 100% ER).

**Table 1:** Patient characteristics

Characteristics	Patients NR (n. 13)	Patients ER (n. 5)
**Median age, years**	64 (44-84)	67.4 (56-77)
**Median follow-up, months (range)**	133.7 (51.6-145.8)	10.4 (0.5-54.2)
**Gender: Male/Female (%)**	10/3 (76.9/23.1)	5/0 (100/0)
**Ever smokers: Yes/No (%)**	12/1 (92.3/7.7)	5/0 (100/0)
**Stage: IA/1B (%)**	8/5 (61.5/38.5)	1/4 (20/80)

### Data analysis and real time Q-PCR validation

**Figure 1:**
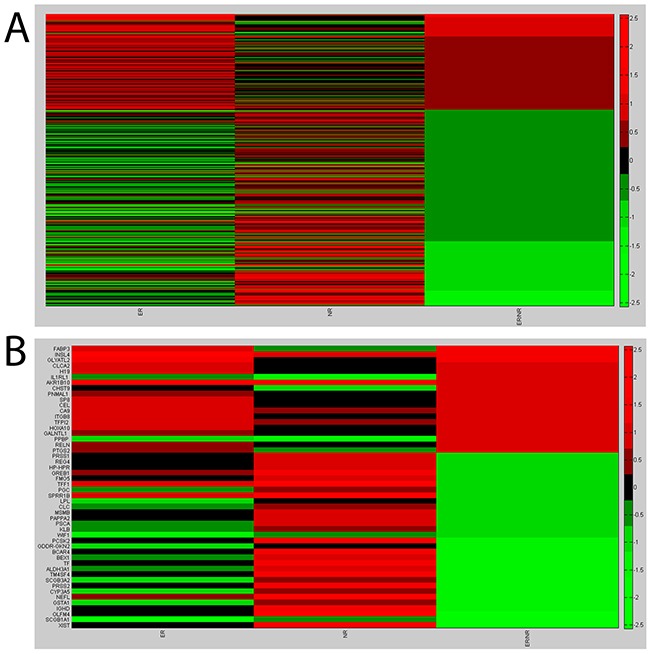
Microarray results **A.** Heat map plot for gene expression ratio- the (log10) of ER vs Normal, NR vs Normal and ER vs NR. **B.** Heat map plot for the selected genes: lower (0.10) and upper (0.90) quartiles of the distribution of the logarithm of the fold of ER versus NR.

**Figure 2:**
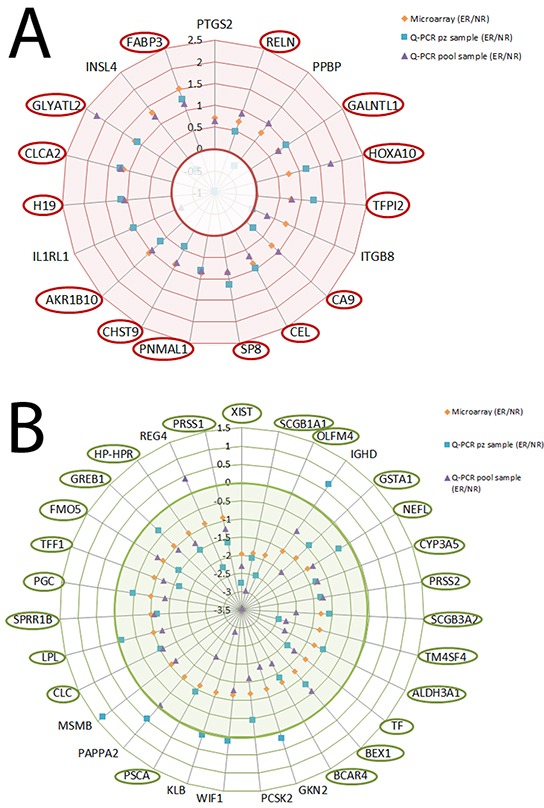
Validation experiments for the increased and decreased selected genes (orange diamond), Q-PCR pool (cyan square) and Q-PCR patients (violet triangle) **A.** Logarithm of the ratio ER vs NR for the increased genes. **B.** Logarithm of the ratio ER vs NR for the decreased genes.

Microarray analysis showed a panel of 436 differentially expressed genes for NR vs NL (p<0.01) (232 increased and 204 decreased) and a panel of 342 differentially expressed genes forER vs NL (p<0.01) (179 increased and 163 decreased). The heat map plot for gene expression ratio (the (log10) of ER vs NL, NR vs NL and ERvs NR) is reported in Figure 1A. Based on the lower (0.10) and upper (0.90) quartiles of the distribution of the logarithm of the fold of ER versus NR, we selected 50 genes (19 increased and 31 decreased) shown in Figure 1B (additional data are available in Supplementary Table S1 and Table S2). We validated by Q-PCRthe expression levels of 19 increased and 31 decreased genes in three pools and in each individual patient sample. The logarithm of the ratio ER versus NR for the increased and decreased genes is shown in Figures 2A and 2B, respectively.

Eighteen of the 19 increased genes showed a concordance of 94.7% between expression levels of microarray and those of the Q-PCR pool (r=0.32, p=0.004): only the IL1R1 gene showed a different value in the Q-PCR pool than the value of the microarray. Similar results were observed in the decreased genes (30 of the 31, (r=0.36 p<0.001): only the REG4 gene showed a different value in the Q-PCR pool than the value of the microarray.

Fourteen of the 19 increased genes showed a concordance of 73.6% between expression levels of microarray and those of the patient Q-PCR (r=0.29, p=0.36): 4 genes (PPBP, ITGB8, IL1RL1, INSL4) showed a different value in the patient sample Q-PCR than that ofthe microarray. Twenty-five of the 31 decreased genes showed a concordance of 80.6% between expression levels of microarray and those of the patient Q-PCR (r=0.26, p=0.24): six genes (IGHD, GKN2, WIF1, KLB,PAPPA2, MSMB) showed a different value in the patient sample Q-PCR thanthe value of the microarray.

### Computational analysis

The selected 14 increased and 25 decreased genes were used to perform a computational gene network analysis. The goal of computational analysis is to select a few key genes from the starting group that are relevant in the activated and inhibited gene networks.

**Figure 3:**
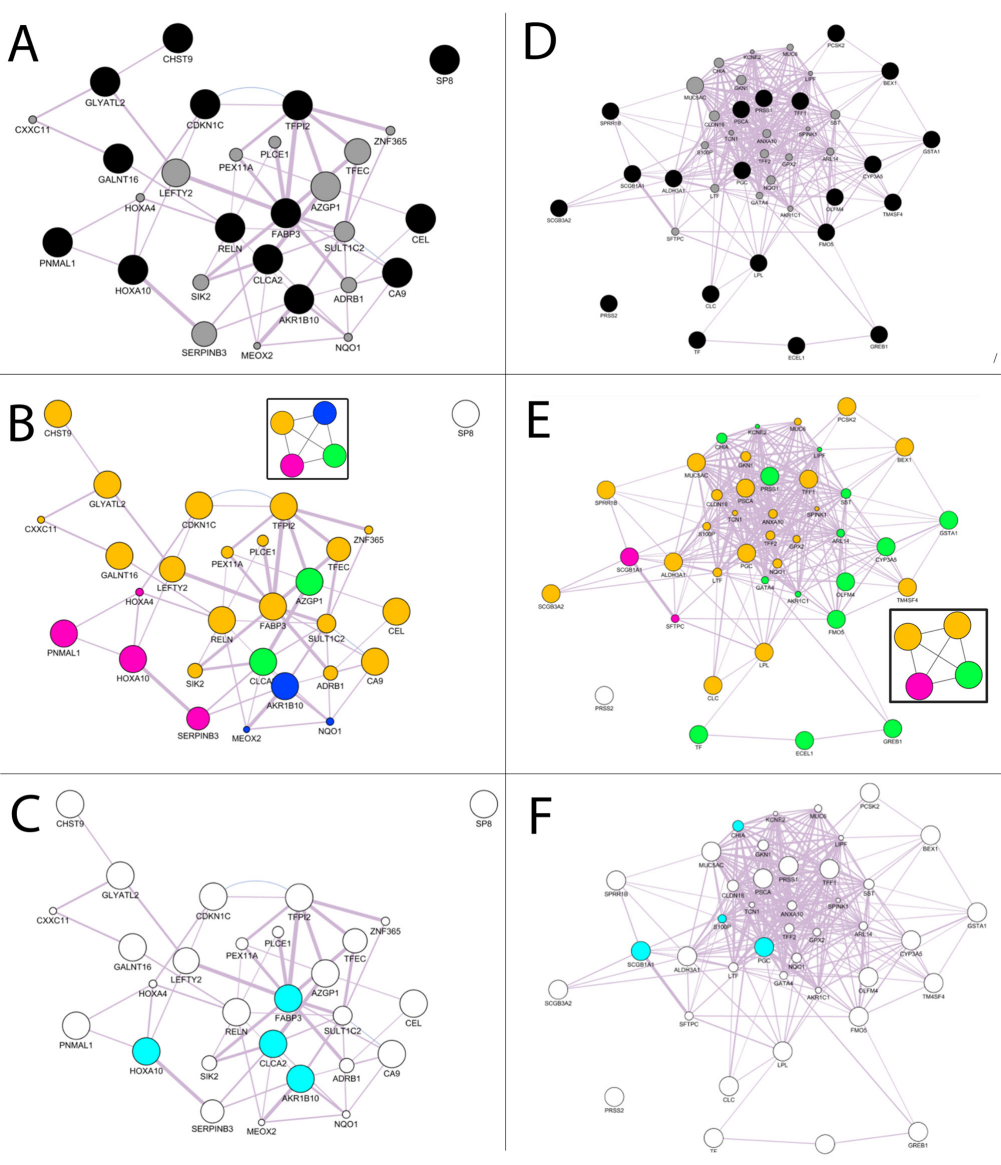
Computational analysis for increased and decreased genes **A.** GeneMANIA networks of validated genes **B.** Community Landscape Analysis obtained with ModuLand plug-in. The four modules were plotted in the graph using a different color for each groupof nodes in the same module. **C.** Key nodes that predict the function of all four modules. **D.** GeneMANIA networks of validated genes **E.** Community Landscape Analysis obtained with ModuLand plug-in. The five modules were plotted in the graph using a different color for each groupof nodes in the same module. **F.** Key nodes that predict the function of all five modules.

The activated gene network was generated with GeneMania tools using as query input the 14 increased genes. Figure 3Ashows the GeneMania network output- obtained setting as inputs: the human organism data, the query gene-based as weighting method, suggestedwhen the input gene list contains more than 5 genes, and co-expression,pathways and predicted as network interactions [10]. The black nodes are the input genes (H19 gene symbol was replaced with the alias CDKN1C gene symbol), the grey nodes are the recommended nodes (the dimensions are proportional to the GeneMania network score) and theviolet link thickness is related to the weight of co-expression interaction inferred from the public data and used in the network generation algorithms [11].

The GeneMania network was elaborated with the ModuLand plug-in that provides an algorithm for determining extensively overlapping network modules [12]. Figure 3B shows the four modules generated for the increased GeneMania network. The nodes in the same module have the same color. ModuLand also identifies several hierarchical layers of modules, where meta-nodes of the higher hierarchical layer represent modules of the lower layer. The tool assigned module cores, which predict the function of the whole module, and determine key nodes that connect two or multiple modules. For the activated gene network the key nodes were HOXA10, CLCA2, AKR1B10, FABP3 (Figure 3C).

As for the increased genes, we applied the same steps as those used for the decreased genes (XIST gene symbol was replaced with the alias ECEL1 gene symbol). Figure 3D shows the GeneMania output. In this case there are yellow links that arethe predicted interactions not proposed by the recommender systems in increased genes. We applied the ModuLand algorithm to the decreased GeneMania network and five modules were obtained (Figure 3E). The selected genes as the central nodes that best predict the function of the five modules obtained with ModuLand were: SCGB1A1, PGC, CHIA and S100P (Figure 3F). CHIA and S100P were not included inthe selected decreased genes but are proposed by the GeneMania recommender systems that integrate the query input information with the public [13]. The same methodology appliedto the increased genes was reproduced to select the validated decreasedgenes using community centrality score [14]. CHIA and S100P were replaced with TFF1, PSCA, SPRR1B and PRSS1 that were the validated genes with the highest value of the score for their module. The 4 increased and 6 decreased genes with their functions are shown in Table 2.

**Table 2:** Function of selected genes

Gene Name	Expression	Functions	References
**HOXA10**	**Increased**	Homebox A10 (HOXA10) is part of the A cluster, on chromosome 7, of the class of transcription factorscalled homebox genes. It encodes a DNA-binding transcription factor that may regulate gene expression, morphogenesis, and differentiation. More specifically, it may function in fertility, embryo viability, and regulation of hematopoietic lineage commitment	[26]
**CLCA2**	**Increased**	The protein encoded by Chloride channel accessory 2 (CLCA2) gene, on chromosome 1, belongs to the calcium sensitive chloride conductance protein family. Since this protein is expressed predominantly in trachea and lung, it is suggested to play a role in the complex pathogenesis of cystic fibrosis. It may also serve as adhesion molecule for lung metastatic cancer cells, mediating vascular arrest and colonization, and furthermore, it has beenimplicated to act as a tumor suppressor gene for breast cancer.	[47] [48]
**AKR1B10**	**Increased**	Aaldo-keto reductase family 1,member B10 (AKR1B10) gene encodes a member of the aldo/keto reductase superfamily, which consists of more than 40 known enzymes and proteins. It is highly expressed in lung, adrenal gland, small intestine and colonand may play an important role in liver carcinogenesis.	[49]
**FABP3**	**Increased**	The intracellular fatty acid-binding proteins (FABPs) belong to a multigene family. FABPs are divided into at least three distinct types, hepatic-, intestinal- and cardiac-type. They participate in the uptake, intracellular metabolism and/or transport of long-chain fatty acids, in the modulation of cell growth and proliferation. FABP3 gene contains four exons and its function is to arrest growth of mammary epithelial cells. This gene is acandidate tumor suppressor gene for human breast cancer.	[50]
**SCGB1A1**	**Decreased**	Secretoglobin, family 1A, member 1(SCGB1A1) gene encodes a member of the secretoglobin family of small secreted proteins. The encoded protein has been implicated in numerous functions including anti-inflammation, inhibition of phospholipase A2 and the sequestering of hydrophobic ligands. Defects inthis gene are associated with a susceptibility to asthma	[51]
**PSCA**	**Decreased**	Prostate stem cell antigen (PSCA) gene encodes a glycosylphosphatidylinositol-anchored cell membrane glycoprotein. In addition to being highly expressed in the prostate it is also expressed in the bladder, placenta, colon, kidney and stomach. This gene is up-regulated in a large proportion of prostatecancers and is also detected in cancers of the bladder and pancreas. The function of PSCA in tumor biology and the regulatory mechanism of PSCA expression still remains unknown.	[30]
**PGC**	**Decreased**	Progastricsin (pepsinogen C) (PGC) gene encodes an aspartic proteinase that belongs to the peptidase family A1. The encoded protein is a digestive enzyme that is produced inthe stomach and constitutes a major component of the gastric mucosa. This protein is also secreted into the serum. This protein is synthesized as an inactive zymogen converted into its active mature format low Ph.	[52]
**PRSS1**	**Decreased**	Protease, serine, 1 (trypsin 1) (PRSS1) gene encodes a trypsinogen, which is a member of the trypsin family of serine proteases. This enzyme is secreted by the pancreas and cleaved to its active form in the small intestine. This gene and severalother trypsinogen genes are localized to the T cell receptor beta locuson chromosome 7.	[53]
**TFF1**	**Decreased**	Trefoil factor 1 (TFF1) gene is a members of the trefoil family. They are stable secretory proteins expressed in gastrointestinal mucosa. Their functions are not defined, but they may protect the mucosa from insults, stabilize the mucus layer,and affect healing of the epithelium. This gene, which is expressed in the gastric mucosa, has also been studied because of its expression in human tumors. This gene and two other related trefoil family member genes are found in a cluster on chromosome 21.	[54]
**SPRR1B**	**Decreased**	Small proline-rich proteins (SPRRs) multi-gene family maps on chromosome 1. Their expression is highin epithelia of oral tissues such as tongue, esophagus and stomach, in contrast to external dry epithelia, such as skin. It has a role during squamous differentiation of skin and respiratory epithelial cells. Moreover, SPRR1 is expressed in squamous tumors of the lung. However, its role in non-squamous cells is largely unknown; it seems that it alsooccurs in non-squamous tissues and cell lines. This protein family is an important component of the cornified cell envelope, a structure formed beneath the plasma membrane of squamous differentiated cells by extensive cross-linking of several proteins.	[55], [56], [57]

### Independent validation set by Q-PCR

**Figure 4:**
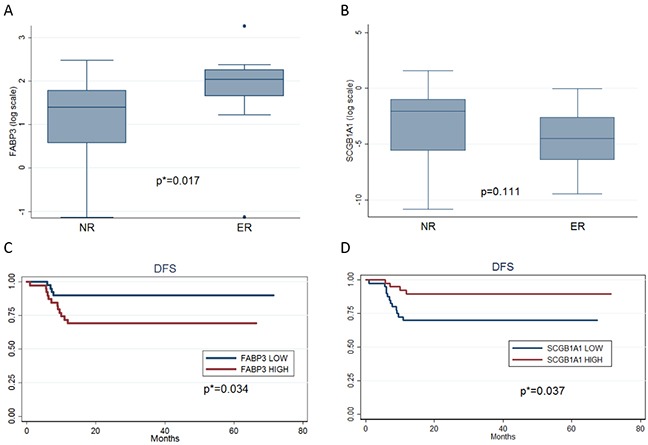
Box plots and Kaplan-Meier estimates for disease-free survival (DFS) for an independent patient population **A.** Box plot for the logarithm of FABP3 gene expression for NR and ER patients. **B.** Box plot for the logarithm of SCGB1A1 gene expression for NR and ER patients. **C.** Kaplan-Meier estimates for disease-free survival (DFS) according to lowand high FABP3 expression with respect to the mean of this gene expression in the study population. **D.** Kaplan-Meier estimates fordisease-free survival (DFS) according to low and high SCGB1A1 expression with respect to the mean of this gene expression in the studypopulation.

To further explore the impact of increased or decreased genes on disease free survival (DFS) we selected FABP3 (increased gene) and SCGB1A1 (decreased gene) as having the two highest GeneMania discriminat network scores (Supplementary Table S3) [13]. They were evaluated by Q-PCR analysis on an independent study set of 79ADs stage I samples. Of these patients, 63 were in NR with a median DFSof 31.7 months (range 12.1-71.4), while the remaining 16 were in ER with a median DFS of 7.4 months (range 0.9-11.8). Median age was 67 years (range 38-81) for NR patients and 68 years (55.0-75.5) for ER patients; most of them were male (66.7% NR, 62.5% ER) and smokers (69.8%NR, 93.8% ER) (Table 3 panel A). The box plots of mRNA expressions of FABP3 and SCGB1A1 in NR and ER groups are shown in Figure 4A and 4B,respectively. High FABP3 expressions were associated with ER patients (p=0.017) with respect to those in NR, while low SCGB1A1 expressions were showen in ER patients as compared to those in NR with border line significance (p=0.111). At univariate analysis, high FABP3 expression and low SCGB1A1 expression were independent predictors of shorter DFS (P=0.034, HR=3.39, CI 95% 1.09-10.52, p=0.037, HR=0.29 CI 95% 0.09-0.92,respectively), as reported in Table 3 panel B and Figure 4C and 4D, respectively.

**Table 3:** Independent patient sample: patient characteristics (Panel A) and disease free survival with Cox model for the selected genes (Panel B)

Panel A		
Characteristics	Patients NR (n. 63)	Patients ER (n.16)
**Median age, years**	67.0 (38-81)	68.0 (55.0-75.5)
**Median DFS, months (range)**	31.7 (12.1-71.4)	7.4 (0.9-11.8)
**Median follow-up, months (range)**	37.2 (12.1-71.4)	9.4 (0.9-44.6)
**Gender: Male/Female (%)**	42/21 (66.7/33.3)	10/6 (62.5/37.5)
**Ever smokers: Yes/No (%)**	44/19 (69.8/30.2)	15/1 (93.8/6.2)
**Stage: IA/1B (%)**	42/21 (66.7/33.3)	8/8 (50.0/50.0)

Panel B				
Variables	HR	95% CI		p
Gender (M/F)	1.16	0.42	3.19	0.775
Age	1.00	0.94	1.06	0.869
Stage (IA/IB)	1.87	0.70	4.99	0.209
FABP3 (High/low)	3.39	1.09	10.52	**0.034**
SCGB1A1(High/low)	0.29	0.09	0.92	**0.037**

## DISCUSSION

The identification of new genes involved in the risk of early relapse of radically resected lung AD patients could assist intheir clinical management. Current evidence supports adjuvant cisplatin-based chemotherapy (four cycles) as standard of care in stagesII and III radically resected NSCLC, while most stage IA and IB patients receive only surgical resection. As a significant proportion ofstage I patients will relapse within 3 years, identification of early-stage patients with a poor prognosis could assist in selecting theappropriate candidates for adjuvant therapy. Several genomic and proteomic approaches have been made to identify signatures that can moreaccurately stratify NSCLC patients [15].

To our knowledge this is the first study that combines high throughput gene expression and computational analysis to identify stage I radically resected patients with an increased risk of relapse. This study proposes a new overall methodology to identify genesthat are relevant in the prediction of disease relapse starting from anRNA sample pool of patients with homogenous stage (only stage I) and histopathological type (ADs). Previous gene profiling studies [16–18]used non-homogenous patient stages and histotypes and a large number ofgenes were selected to predict outcomes that are not easily applicable in the clinical setting. The methodology of our study by computational and bioinformatic analysis allowed us to select a few genes that discriminated for risk of relapse and can be used in clinical practice.

Microarray technology is commonly used in basic medical and biological research but has not yet been used for routine analysis in clinical practice. Today, various microarray platforms are used for generating RNA expression profiling and there is no gold standard, which probably are two possible explanations of conflicting results [16–19]. Real-time Q-PCR is considered the—gold standard' for gene expression analysis and is commonly used for validation of microarray results [20]. In our study, using Affymetrix microarray technology, we were able to identify a number of genes that were subsequently validated using real time Q-PCR, both in the pools and in individual RNA samples of selected patients in ER and NR disease. Validation of expression levels of increased and decreased genes by Q-PCR in the pools showed a concordanceof 94.7% with respect to expression levels of microarray data (p<0.001). Due to the variability of gene expression among the RNA samples, as observed in another study [21],the validation of expression levels of increased and decreased genes byQ-PCR in the individual patients showed a lower concordance (73.6% for increased and 80.6% for decreased genes) with respect to the values of microarray expression levels (p<0.001).

Using computational analysis we were able to identify 4 increased and 6 decreased genes as the central nodes that best predict the function of the five modules obtained with ModuLand. The increased genes HOXA10, CLCA2, AKR1B10 and FABP3 were all present inthe validated genes, while for the decreased genes only SCGB1A1 and PGCwere in the validated gene list. CHIA and S100P were proposed by the GeneMania recommender systems and related to the centrality score to TFF1, PSCA, SPRR1B and PRSS1 that were in the selected list. Our ER patients presented a specific high expression of HOXA10, CLCA2, AKR1B10 and FABP3 (increased genes) and a low expression of SCGB1A1, PGC, TFF1, PSCA, SPRR1B and PRSS1 (decreased genes) with respect to NR patients.

As FABP3 and SCGB1A1 genes had the highest discriminant network score in the computational analysis, we used an independent population of stage I Ads to assess their prognostic significance on DFS. We demonstrated that high FABP3 expression and low SCGB1A1 expression was associated with a worse DFS. In NSCLC the prognostic role of FABP3 is not known, while in gastric cancer, high expression of heart-type FABP protein is associated with increased tumoraggressiveness, metastasis and poor prognosis [22]. Interestingly, in NSCLC cell lines, increased expression of heart-FABP appears to have a role in predicting response to gefitinib [23]. Davidson's study, using a methodology similar to that of our study, identified sets of genes that were differentially expressed in leiomyosarcoma (LMS) than in endometrial stromal sarcoma. Among these genes, FABP3 was predominantly overexpressed in LMS [24].

SCGB1A1 can be used as a marker of precancerous progression in the bronchial epithelium since it is involved in the process of damage repair and xenobiotic metabolism; when decreased it isoften associated with neoplastic transformation. As in our study, lowerlevels of this protein were associated with a worse clinical outcome ina population at high risk for lung cancer. The expression of SCGB1A1 also appears to be inversely correlated to regression of bronchial dysplasia and improvement in sputum cytometry assessment in smokers withhigh lung cancer risk. [25]

HOXA10 has been reported to be increased in human lung cancer cell lines and lung tumor tissues with respect to normal lung [26].

CLCA2 has a significant effect on cell invasion and the metastatic process and its expression is increased as a result of cell damage. It regulates the apoptotic pathway, senescence and carcinogenicity trough p53 [27]. Moreover, the CLCA2 gene has been shown to act as a tumor suppressor in breast and colon cancer, where it is often decreased. On the contrary, overexpression of CLCA2 has been reported to be specifically associated with NSCLC.

AKR1B10 has been proposed as a promising new diagnostic marker in NSCLC of smokers since it is often overexpressed inmoderate or poorly differentiated NSCLC (in 84% of squamous cell carcinomas and in 29% of adenocarcinomas), mostly in males and smokers [28].

Among decreased genes, PSCA has a role in signal transduction and in cell growth regulation. In a gastric cancer cell line it has been shown to have cell growth inhibitory activity [29] while in gallbladder adenocarcinoma it seems to be overexpressed and to correlate with decreased survival [30]. The expression status of PSCA in cancer cells appears to depend on the epithelium of their origin [31]. In NSCLC its expression is negatively correlated with prognosis [32].

The PGC gene has a general role in suppressing tumordevelopment and several studies reported that low expression of PGC protein was closely related to poor differentiation and unfavorable survival in patients with breast, prostate, gastric and ovarian cancers [33–35].

PRSS1 gene expression seems to induce apoptosis and to promote spontaneous pancreatitis [36] and its role is not known in NSCLC.

TFFs is overexpressed in prostate, breast and lung AD and seems to be an indicator of worse prognosis. In lung adenocarcinoma, TFF1 gene expression is significantly associated with larger tumor size and acinar subtype [37]. Foekens et al. showed that decreased TFF1 in early breast cancer is an important variable for the identification of patients at high risk for recurrence and death [38]. In gastric tumors a decrease of TFF1 significantly reduced the apoptosis of the cell lines and facilitated their proliferation [39].

The SPRR1B gene has a role in the transition of cells to G0 and may disrupt the normal progression to mitosis resulting in changes in ploidy. SPRR1B is a potential biomarker for bronchial malignant transformation since its expression is markedly reduced duringtumor progression and its loss induces an irreversible malignant transformation [40].

In conclusion, our results indicate that it is possible to define, through gene expression and computational analysis, acharacteristic gene profiling of ER patients with an increased risk of disease relapse. We also validated FABP3 and SCGB1A1 genes as independent prognostic factors of worse DFS. This may pave the way to more effective patient selection for adjuvant studies and possibly yieldnovel therapeutic targets with potential for drug development.

## MATERIALS AND METHODS

### Overall workflow method

**Figure 5:**
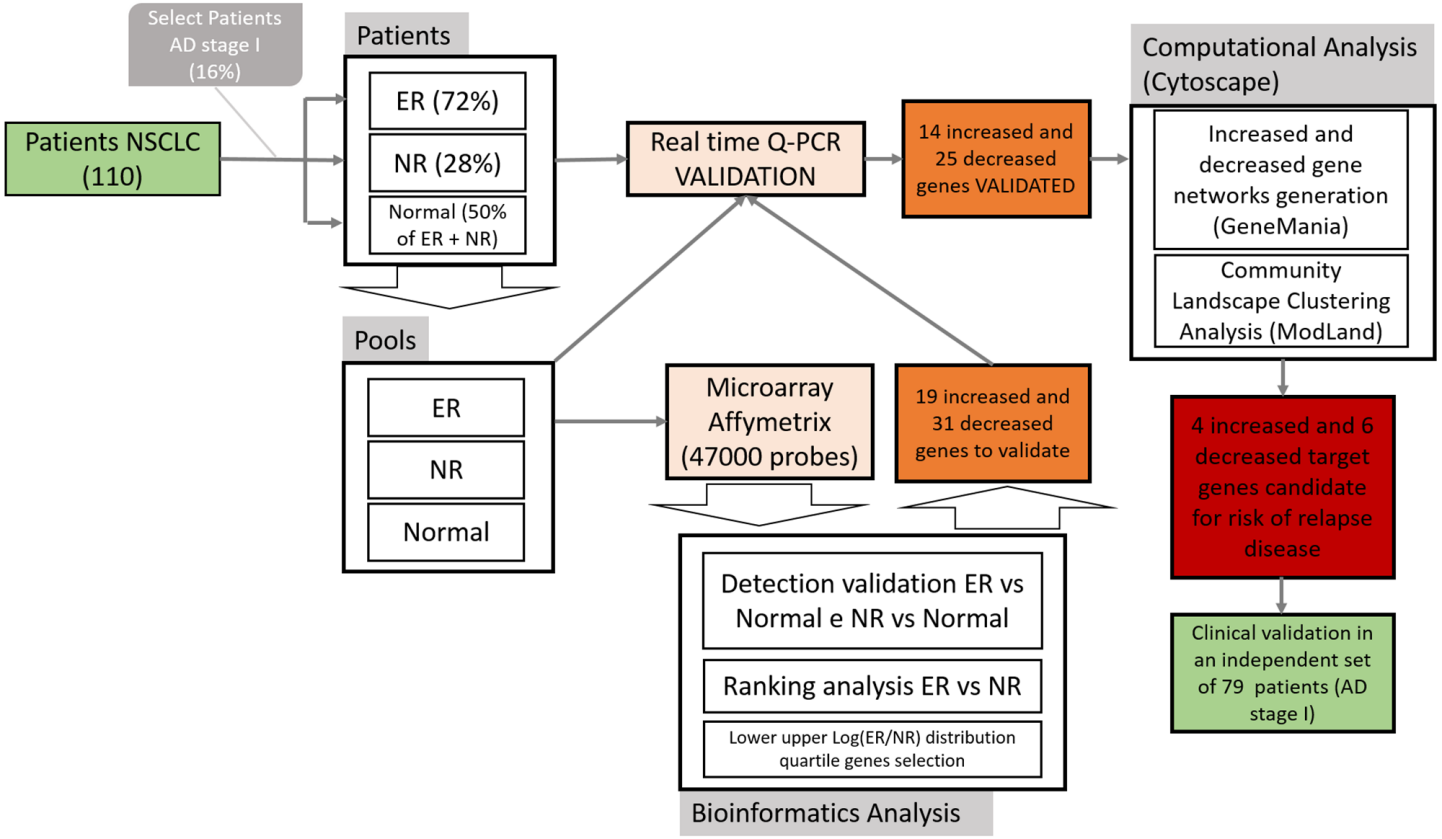
Study flowchart

We introduced a workflow diagram to synthetize the overall methodology applied in this paper (Figure 5). The goal of the study was to select a subgroup of genes relevant for risk of early relapse in NSCLC patients. From a database of consecutive resected NSCLC patients we identified stage I adenocarcinomas. The samples of patients in ER, NR and normal lung (NL) tissue were then identified. Three RNA pools were created, one for each group (ER, NR, NL) that were the input of the Microarray Affymetrix technology. The 47000 probes output for ER, NR, e NL were analysed using bioinformatic tools: detection validation analysis (ER vs NL and NR vs NL), ranking analysis (ER vs NR) and log distribution fold changes quartiles analysis(ER vs NR). These analyses were used to select 19 increased and 31 decreased genes. A real time quantitative-PCR (Q-PCR) validation step for the selected genes was performed using single patient samples and three RNA pools. We selected the 14 increased and 25 decreased genes that showed a concordance between microarray and Q-PCR expression levelsin patients and pools. These genes were used as input for computationalanalysis based on GeneMania and ModLand tools of Cytoscape. We obtained4 increased and 6 decreased genes that could be used as a genetic signature to predict early relapse. Furthermore, an independent set of 79 DCs stage I samples was used to validate selected genes by Q-PCR.

### Patient selection

As illustrated in Figure 5, using aprospective tissue banking of 110 consecutive resected NSCLC patients at S. Maria della Misericordia Hospital in Perugia-Italy, we selected frozen specimens of patients with stage I lung adenocarcinoma [2], [41].

Patients and their corresponding tissues were divided as follow: 1) no relapse (NR) based on the estimated risk of recurrence of these patients [42]; 2) early relapse (ER) disease relapse within a year from surgery; 3) normallung (NL) tissue taken from distant or contralateral lung from both groups (1 and 2) of patients. From these three groups of patients, threeRNA pools were created: pool ER, pool NR, pool NL.

Tissues were stored in a freezer at −80°C in a solution containing RNAlater (Qiagen S.p.A., Milan, Italy). Samples had to contain at least 50% tumor cells to be eligible for microarray analysis as determined by one reference pathologist (B.G.) on adjacent separate sections.

The study was approved by the local Ethics Committeeand was conducted in accordance with ethical principles of the latest version of the Declaration of Helsinki. Written informed consent for gene expression analyses was obtained from each patient entering the study.

### RNA extraction, microarray and Q-PCR

Total RNA was extracted from frozen tissues after thawing and homogenizing by IKA Ultra-Turrax and QIAzol Lysis Reagent. RNA was extracted with the phenol chloroform method. From the aqueous phase, RNA was automatically purified by BioRobot EZ1using EZ1 RNA Universal Tissue kit instrument according to the manufacturer's instructions (Qiagen S.p.A., Milan, Italy). RNA was eluted in 50 μl of RNase-free water and stored at −80°C until use. The quality, integrity and quantity of the total RNA was evaluated on Experion™ Bioanalyzer (Biorad Technologies, Italy).

To create the three pools, a concentration of 1 γ for each sample belonging to each pool was used. The concentration of the three pools was assessed and the volume of each pool was calculated to obtain the required concentration (5 γ) for the Microarray. We compared gene expression profiling from pool-NL and cancer specimens from pool-NR and pool-ER, using Affimetrix human microarray HG-U133Plus 2.0 that evaluates the expression levels of more than 47,000 human transcripts and variants, including 38,500 well-characterized genes in asingle experiment. We used reverse transcription PCR (RT-PCR) to createfrom RNA complementary DNA (cDNA) using random primer technologies. Expression of cDNA for increased and decreased genes was measured in thethree pools (NR, ER, NL) and in each of the lung tissues of corresponding patients by real-time Q-PCR using SybrGreen Gene Expression Assays on Stratagene Mx3000P QPCR System. Premier Biosoft Beacon Designer was used to design the primers. The housekeeping gene was the HPRT (Hypoxantin PhosphoRibosyl Transferase); the calibrator wasthe RNA pool of normal lung tissue. All runs included a calibrator sample and a one no-template control, and all samples were measured in triplicate. Relative quantification was carried out using the 2^−ΔΔCt^ method using HPRT as reference gene [43]. Increased and decreased genes were presented using radar plot for the logarithm of the rate of ER versus NR. The microarray data, pool and patient Q-PCR mean of the gene expressions were compared with the 0 of the logarithm of the ER and NR ratio. The correlation and the concordance were tested with Kendall's correlations with a poisson regression.

### Bioinformatics analysis

Filtering criteria were applied to select significant genes. A one-sided Wilcoxon's Signed Rank test was the statistical method used to calculate the detection p-value (<0.01) that reflects the significance of the differences between perfect match intensity and mismatch intensity for each probe pair of the microarray. Next, a ranking analysis of microarray data based on F-statistic [44] (*P*< 0.01) was used to identify statistically significant genes from ERversus NR experiments. Agilent SIGNET and Matlab software were used to perform bio-informatic analysis.

The increased and decreased genes to be analyzed in the validate setting were chosen considering the logarithm of the ratio between ER and NR and applying the lower and the upper quantiles (0.10 and 0.90) of the distribution.

### Real time Q-PCR in the independent validation set

An independent validation set of 79 ADs patients collected at the S. Maria della Misericordia Hospital (Perugia, Italy) from 2008 to 2014 was used to further investigate, using Q-PCR, the impact of increased or decreased genes on disease- free survival (DFS). We evaluated FABP3 (increased gene) and SCGB1A1 (decreased gene) representing the network central nodes. Quantification of mRNA expression levels of two genes, were performed by real-time one-step RT-PCR using QuantiFast assay (Qiagen, Milan, Italy).

The results were compared considering β-actin (internal reference gene) and the pool of normal tissues (calibrator) using the 2^−ΔΔCt^method. DFS and the 95% confidence intervals (CIs) were evaluated by the Kaplan–Meier method comparing the different groups by log-rank test. The Cox proportional hazards model was used to evaluate the prognostic role of each single parameter studied on DFS, in univariate analyses.

### Gene network computational analysis

Gene network analysis was performed to evaluate the network representation of the molecular relationships between increased and decreased selected genes in ER vs NR. GeneMANIA, a Cytoscape plug-in, was used to predict increased and decreased gene interactions and to expand the networks with functionally similar genes, using available genomics and proteomics data [10]. Networks were generated using information derived from the co-expression, pathways and predicted categories.

Key network nodes were identified through the analysis of network properties including connectedness and centrality. We used the ModuLand algorithm, implemented as plug-in of Cytoscape, to analyze the properties of the activated or increased and inhibited or decreased networks that were obtained using GeneMANIA. The ModuLand algorithm produced clusters (or modules) represented by key network nodes [12]. The function of these centralnodes best predicts the function of the module it represents. The central nodes are also likely to represent the key functional elements of the overall network and therefore can be used to prioritise future work [45], [46].

## SUPPLEMENTARY TABLES





## References

[1] Jemal A, Siegel R, Ward E, Murray T, Xu J, Smigal C, Thun MJ (2006). Cancer Statistics, 2006. CA Cancer J Clin.

[2] Rami-Porta R, Crowley JJ, Goldstraw P (2009). The revised TNM staging system for lung cancer. Ann Thorac Cardiovasc Surg.

[3] Pignon J-P, Tribodet H, Scagliotti G V, Douillard J-Y, Shepherd FA, Stephens RJ, Dunant A, Torri V, Rosell R, Seymour L (2008). Lung adjuvant cisplatin evaluation: a pooled analysis by the LACE Collaborative Group. J Clin Oncol.

[4] Strauss GM (2006). Management of early-stage lung cancer: past, present, and future adjuvant trials. Oncology (Williston Park).

[5] Chen H-Y, Yu S-L, Chen C-H, Chang G-C, Chen C-Y, Yuan A, Cheng C-L, Wang C-H, Terng H-J, Kao S-F (2007). A five-gene signature and clinical outcome in non-small-cell lung cancer. N Engl J Med.

[6] Potti A, Mukherjee S, Petersen R, Dressman HK, Bild A, Koontz J, Kratzke R, Watson MA, Kelley M, Ginsburg GS (2006). A genomic strategy to refine prognosis in early-stage non-small-cell lung cancer. N Engl J Med.

[7] Raponi M, Zhang Y, Yu J, Chen G, Lee G, Taylor JMG, MacDonald J, Thomas D, Moskaluk C, Wang Y (2006). Gene expression signatures for predicting prognosis of squamous cell and adenocarcinomas of the lung. Cancer Res.

[8] Bhattacharjee a, Richards WG, Staunton WG (2001). Others. Classification of human lung carcinomas by mRNA expression profiling reveals distinct adenocarcinoma subclasses. Proc Natl Acad Sci.

[9] Lau SK, Boutros PC, Pintilie M, Blackhall FH, Zhu CQ, Strumpf D, Johnston MR, Darling G, Keshavjee S, Waddell TK (2007). Three-gene prognostic classifier for early-stage non-small-cell lung cancer. J Clin Oncol.

[10] Montojo J, Zuberi K, Rodriguez H, Kazi F, Wright G, Donaldson SL, Morris Q, Bader GD (2010). GeneMANIA cytoscape plugin: Fast gene function predictions on the desktop. Bioinformatics.

[11] Warde-Farley D, Donaldson SL, Comes O, Zuberi K, Badrawi R, Chao P, Franz M, Grouios C, Kazi F, Lopes CT (2010). The GeneMANIA prediction server: biological network integration for gene prioritization and predicting gene function. Nucleic Acids Res.

[12] Szalay-Beko M, Palotai R, Szappanos B, Kovács IA, Papp B, Csermely P (2012). ModuLand plug-in for Cytoscape: Determination of hierarchical layers of overlapping network modules and community centrality. Bioinformatics.

[13] Bianconi F, Patiti F, Baldelli E, Crin L, Valigi P (2015). An approach for optimally extending mathematical models of signaling networks using omics data. EMBC.

[14] Farkas IJ, Korcsmáros T, Kovács IA, Mihalik Á, Palotai R, Simkó GI, Szalay KZ, Szalay-Beko M, Vellai T, Wang S (2011). Network-based tools for the identification of novel drug targets. Sci Signal.

[15] Meyerson M, Carbone D (2005). Genomic and proteomic profiling of lung cancers: lung cancer classification in the age of targeted therapy. J Clin Oncol.

[16] Irizarry RA, Warren D, Spencer F, Kim IF, Biswal S, Frank BC, Gabrielson E, Garcia JGN, Geoghegan J, Germino G (2005). Multiple-laboratory comparison of microarray platforms. Nat Methods.

[17] Larkin JE, Frank BC, Gavras H, Sultana R, Quackenbush J (2005). Independence and reproducibility across microarray platforms. Nat Methods.

[18] Pawitan Y, Michiels S, Koscielny S, Gusnanto A, Ploner A (2005). Gene expression False discovery rate, sensitivity and sample size for microarray studies. Bioinformatics.

[19] Allison DB, Cui X, Page GP, Sabripour M (2006). Microarray data analysis: from disarray to consolidation and consensus. Nat Rev Genet.

[20] Mackay IM, Arden KE, Nitsche A (2002). Real-time PCR in virology. Nucleic Acids Res.

[21] Loi S, Haibe-kains B, Desmedt C, Wirapati P, Lallemand F, Tutt AM, Gillet C, Ellis P, Ryder K, Reid JF (2008). Predicting prognosis using molecular profiling in estrogen receptor-positive breast cancer treated with tamoxifen. BMC Genomics.

[22] Hashimoto T, Kusakabe T, Sugino T, Fukuda T, Watanabe K, Sato Y, Nashimoto A, Honma K, Kimura H, Fujii H (2004). Expression of heart-type fatty acid-binding protein in human gastric carcinoma and its association with tumor aggressiveness, metastasis and poor prognosis. Pathobiology.

[23] Okano T, Kondo T, Fujii K, Nishimura T, Takano T, Ohe Y, Tsuta K, Matsuno Y, Gemma A, Kato H (2007). Proteomic signature corresponding to the response to gefitinib (Iressa, ZD1839), an epidermal growth factor receptor tyrosine kinase inhibitor in lung adenocarcinoma. Clin Cancer Res.

[24] Davidson B, Abeler VM, Hellesylt E, Holth A, Shih I-M, Skeie-Jensen T, Chen L, Yang Y, Wang T-L (2013). Gene expression signatures differentiate uterine endometrial stromal sarcoma from leiomyosarcoma. Gynecol Oncol.

[25] Chen J, Lam S, Pilon A, McWilliams A, Macaulay C, Szabo E (2008). Higher levels of the anti-inflammatory protein CC10 are associated with improvement in bronchial dysplasia and sputum cytometric assessment in individuals at high risk for lung cancer. Clin Cancer Res.

[26] Abe M, Hamada J-I, Takahashi O, Takahashi Y, Tada M, Miyamoto M, Morikawa T, Kondo S, Moriuchi T (2006). Disordered expression of HOX genes in human non-small cell lung cancer. Oncol Rep.

[27] Tanikawa C, Nakagawa H, Furukawa Y, Nakamura Y, Matsuda K (2012). CLCA2 as a p53-inducible senescence mediator. Neoplasia.

[28] Kang M-W, Lee E-S, Yoon SY, Jo J, Lee J, Kim HK, Choi YS, Kim K, Shim YM, Kim J (2011). AKR1B10 is associated with smoking and smoking-related non-small-cell lung cancer. J Int Med Res.

[29] Sakamoto H, Yoshimura K, Saeki N, Katai H, Shimoda T, Matsuno Y, Saito D, Sugimura H, Tanioka F, Kato S (2008). Genetic variation in PSCA is associated with susceptibility to diffuse-type gastric cancer. Nat Genet.

[30] Zou Q, Yang L, Yang Z, Huang J, Fu X (2013). PSCA and Oct-4 expression in the benign and malignant lesions of gallbladder: Implication for carcinogenesis, progression, and prognosis of gallbladder adenocarcinoma. Biomed Res Int.

[31] Ono H, Hiraoka N, Lee Y-S, Woo SM, Lee WJ, Choi IJ, Saito A, Yanagihara K, Kanai Y, Ohnami S (2012). Prostate stem cell antigen, a presumable organ-dependent tumor suppressor gene, is down-regulated in gallbladder carcinogenesis. Genes Chromosomes Cancer.

[32] Kawaguchi T, Sho M, Tojo T, Yamato I, Nomi T, Hotta K, Hamada K, Suzaki Y, Sugiura S, Kushibe K (2010). Clinical significance of prostate stem cell antigen expression in non-small cell lung cancer. Jpn J Clin Oncol.

[33] Scorilas A, Diamandis EP, Levesque MA, Papanastasiou-Diamandi A, Khosravi MJ, Giai M, Ponzone R, Roagna R, Sismondi P, López-Otin C (1999). Immunoenzymatically determined pepsinogen C concentration in breast tumor cytosols: an independent favorable prognostic factor in node-positive patients. Clin Cancer Res.

[34] Díaz M, Rodríguez JC, Sánchez J, Sánchez MT, Martín A, Merino AM, Vizoso F Clinical significance of pepsinogen C tumor expression in patients with stage D2 prostate carcinoma. Int J Biol Markers.

[35] Rojo JV, Merino AM, González LO, Vizoso F (2002). Expression and clinical significance of pepsinogen C in epithelial ovarian carcinomas. Eur J Obstet Gynecol Reprod Biol.

[36] Athwal T, Huang W, Mukherjee R, Latawiec D, Chvanov M, Clarke R, Smith K, Campbell F, Merriman C, Criddle D (2014). Expression of human cationic trypsinogen (PRSS1) in murine acinar cells promotes pancreatitis and apoptotic cell death. Cell Death Dis.

[37] Higashiyama M, Doi O, Kodama K, Yokouchi H, Inaji H, Nakamori S, Tateishi R (1994). Prognostic significance of pS2 protein expression in pulmonary adenocarcinoma. Eur J Cancer.

[38] Foekens J a, Rio MC, Seguin P, Van Putten WLJ, Fauque J, Nap M, Klijn JGM, Chambon P (1990). Prediction of relapse and survival in breast cancer patients by pS2 protein status. Cancer Res.

[39] Ge Y, Zhang J, Cao J, Wu Q, Sun L, Guo L, Wang Z (2012). TFF1 inhibits proliferation and induces apoptosis of gastric cancer cells in vitro. Bosn J Basic Med Sci.

[40] Tesfaigzi Y, Wright PS, Belinsky S a (2003). SPRR1B overexpression enhances entry of cells into the G0 phase of the cell cycle. Am J Physiol Lung Cell Mol Physiol.

[41] Beasley MB, Brambilla E, Travis WD (2005). The 2004 World Health Organization classification of lung tumors. Semin Roentgenol.

[42] Arriagada R, Auperin A, Burdett S, Higgins JP, Johnson DH, Le Chevalier T, Le Pechoux C, Parmar MKB, Pignon JP, Souhami RL (2010). Adjuvant chemotherapy, with or without postoperative radiotherapy, in operable non-small-cell lung cancer: two meta-analyses of individual patient data. Lancet.

[43] Livak KJ, Schmittgen TD (2001). Analysis of relative gene expression data using real-time quantitative PCR and the 2(−Delta Delta C(T)) Method. Methods.

[44] Tan Y-D, Fornage M, Xu H (2008). Ranking analysis of F-statistics for microarray data. BMC Bioinformatics.

[45] Kovács IA, Palotai R, Szalay MS, Csermely P (2010). Community landscapes: an integrative approach to determine overlapping network module hierarchy, identify key nodes and predict network dynamics. PLoS One.

[46] Mones E, Vicsek L, Vicsek T (2012). Hierarchy measure for complex networks. PLoS One.

[47] Abdel-Ghany M, Cheng HC, Elble RC, Pauli BU (2002). Focal adhesion kinase activated by beta(4) integrin ligation to mCLCA1 mediates early metastatic growth. J Biol Chem.

[48] Abdel-Ghany M, Cheng HC, Elble RC, Pauli BU (2001). The Breast Cancer??4 Integrin and Endothelial Human CLCA2 Mediate Lung Metastasis. J Biol Chem.

[49] Fukumoto S, Yamauchi N, Moriguchi H, Hironaka M, Ishikawa Y, Kodama T, Nishimura M (2005). Overexpression of the aldo-keto reductase family protein AKR1B10 is highly correlated with smokers' non-small cell lung carcinomas. Clin Cancer Res.

[50] Nevo J, Mattila E, Pellinen T, Yamamoto DL, Sara H, Iljin K, Kallioniemi O, Bono P, Heikkilä P, Joensuu H (2009). Mammary-derived growth inhibitor alters traffic of EGFR and induces a novel form of cetuximab resistance. Clin Cancer Res.

[51] Rawlins EL, Okubo T, Xue Y, Brass DM, Auten RL, Hasegawa H, Wang F, Hogan BLM (2009). The role of scgb1a1+ clara cells in the long-term maintenance and repair of lung airway, but not alveolar, epithelium. Stem Cell.

[52] Gritti I, Banfi G, Roi GS (2000). Pepsinogens: physiology, pharmacology pathophysiology and exercise. Pharmacol Res.

[53] Whitcomb DC (2013). Genetic risk factors for pancreatic disorders. Gastroenterology.

[54] Prest SJ, May FE, Westley BR (2002). The estrogen-regulated protein, TFF1, stimulates migration of human breast cancer cells. FASEB J.

[55] Tesfaigzi J, Carlson DM (1999). Expression, regulation, and function of the SPR family of proteins. A review. Cell Biochem Biophys.

[56] Tesfaigzi J, Wright PS, Oreffo V, An G, Wu R, Carlson DM (1993). A small proline-rich protein regulated by vitamin A in tracheal epithelial cells is induced in lung tumors. Am J Respir Cell Mol Biol.

[57] Steinert PM, Marekov LN (1999). Initiation of assembly of the cell envelope barrier structure of stratified squamous epithelia. Mol Biol Cell.

