# Supplementary material for: Gene identification for risk of relapse in stage I lung adenocarcinoma patients: a combined methodology of gene expression profiling and computational gene network analysis
Source: Oncotarget. 2016 Apr 13;7(21):30561–74. doi: 10.18632/oncotarget.8723 (PMC5058701; doi:10.18632/oncotarget.8723)
Supplement: Supplementary file 1 [file oncotarget-07-30561-s001.pdf]

Gene identification for risk of relapse in stage I lung adenocarcinoma patients: a combined methodology of gene expression profiling and computational gene network analysis

SUPPLEMENTARY TABLES

Supplementary Table S1: Increased genes

RefSeq Transcript ID	Gene Title	Gene Symbol	NR vs NL	ER vs NL	ER vs NR
NM_004102	Fatty acid binding protein 3, muscle and heart	FABP3	-2,54838	12,9746	33,0641
NM_002195	insulin-like 4 (placenta)	INSL4	14,2474	298,881	20,9779
NM_145016	glycine-N-acyltransferase-like 2	GLYATL2	1,02613	15,1925	14,8056
NM_006536	CLCA family member 2, chloride channel regulator	CLCA2	-1,31477	10,8036	14,2042
NM_003856<br>NM_016232	interleukin 1 receptor-like 1	IL1RL1	-28,9963	-2,53838	11,4231
NR_002196	H19, imprinted maternally expressed transcript (non-protein coding)	H19	1,03511	12,2745	11,8582
NM_020299	aldo-keto reductase family 1, member B10 (aldose reductase)	AKR1B10	20,8573	231,874	11,1171
NM_031422	carbohydrate (N-acetylgalactosamine 4-0) sulfotransferase 9	CHST9	-9,34989	1,69468	5,51721
NM_001807	carboxyl ester lipase (bile salt-stimulated lipase	CEL	1,12253<br>1,7046	7,97614<br>11,2865	7,10552<br>6,62119
NM_182700<br>NM_198956<br>NR_024112	Sp8 transcription factor	SP8	1,12714	8,11128	7,19635
NM_002214	integrin, beta 8	ITGB8	1,0153	6,13108	6,03867
NM_001216	carbonic anhydrase IX	CA9	2,0413	12,3794	6,06449
NM_018951<br>NM_153715	homebox A10	HOXA10	1,13382	6,49194	5,72571
NM_020692	UDP-N-acetyl-alpha-D-galactosamine:polypeptide N-acetylgalactosaminyltransferase	GALNTL1	-1,3463	4,22623	5,68979
NM_002704	pro-platelet basic protein (chemokine (C-X-C motif) ligand 7)	PPBP	-36,9476	-6,8792	5,37092
NM_005045<br>NM_173054	Reelin	RELN	-1,41962	3,6411	5,16898
NM_000963	prostaglandin-endoperoxide synthase 2 (prostaglandin G/H synthase and cyclooxyge)	PTGS2	-2,06279	2,45773	5,06977
NM_001122633<br>NM_001122634<br>NM_001875	carbamoyl-phosphate synthetase 1, mitochondrial	CPS1	58,8098	214,566	3,64848
NM_006528	tissue factor pathway inhibitor 2	TFPI2	1,97356	9,83974	4,98578
NM_001103149<br>NM_018215	PNMA-like 1	PNMAL1	-1,54819	4,71708	7,30293

Supplementary Table S2: Decreased genes

See Supplementary File 1

Supplementary Table S3: Discriminant network score for increased and decreased genes

**Genes increased**		**Discriminant network score**
	**FABP3**	**0.70707**
	CLCA2	0.61527
	HOXA10	0.60134
	AKR1B10	0.56628
**Genes decreased**		**Discriminant network score**
	**SCGB1A1**	**0.58448**
	PRSS1	0.56116
	PSCA	0.54840
	PGC	0.52750
	TFF1	0.51642
	SPRR1B	0.51398
