# Supplementary material for: Gene identification for risk of relapse in stage I lung adenocarcinoma patients: a combined methodology of gene expression profiling and computational gene network analysis
Source: Oncotarget. 2016 Apr 13;7(21):30561–74. doi: 10.18632/oncotarget.8723 (PMC5058701; doi:10.18632/oncotarget.8723)
Supplement: Supplementary file 2 [file oncotarget-07-30561-s002.docx]

**Supplementary Table 2. Decreased** **genes**

| **RefSeq Transcript ID** | **Gene Title** | **Gene Symbol** | **NR vs NL** | **ER vs NL** | **ER vs NR** |
| --- | --- | --- | --- | --- | --- |
| NM_145740 | glutathione S-transferase A1 | GSTA1 | 2,17679 | -12,4217 | -27,0394 |
| AK090461 | immunoglobulin heavy constant delta | IGHD | 26,7421 | -1,27835 | -34,1857 |
| NM_006418 | olfactomedin 4 | OLFM4 | 75,9822 | 1,02349 | -74,2384 |
| NM_003357 | secretoglobin, family 1A, member 1 (uteroglobin) | SCGB1A1 | -4,4954 | -368,909 | -82,0636 |
| NR_001564 | X (inactive)-specific transcript (non-protein coding) | XIST | 98,6283 | 1,07365 | -91,8622 |
| NM_006158 | neurofilament, light polypeptide 68kDa | NEFL | 79,5715 | 3,39901 | -23,4102 |
| NM_000777 | cytochrome P450, family 3, subfamily A, polypeptide 5 | CYP3A5 | 2,35182 | -9,8939 | -23,2686 |
| NM_002770 | protease, serine, 2 (trypsin 2) | PRSS2 | 30,1353 | 1,39583 | -21,5895 |
| NM_054023 | secretoglobin, family 3A, member 2 | SCGB3A2 | 2,3601 | -8,89207 | -20,9862 |
| NM_004617 | transmembrane 4 L six family member 4 | TM4SF4 | 21,6645 | 1,10629 | -19,5831 |
| NM_000691 | aldehyde dehydrogenase 3 family, memberA1 | ALDH3A1 | 8,74925 | -2,23721 | -19,5739 |
| NM_001063 | Transferrin | TF | 28,1611 | 1,49269 | -18,866 |
| NM_018476 | brain expressed, X-linked 1 | BEX1 | 9,14837 | -2,03343 | -18,6026 |
| NR_024049  //  NR_024050 | breast cancer anti-estrogen resistance 4 | BCAR4 | 16,3695 | -1,03221 | -16,8968 |
| NM_182536 | gastrokine 2 | GKN2 | 1,64 | -10,2673 | -16,8384 |
| NM_007191 | WNT inhibitory factor 1 | WIF1 | -1,8706 | -26,3502 | -26,3502 |
| NM_005672 | prostate stem cell antigen | PSCA | 6,34762 | -2,08815 | -13,2548 |
| NM_175737 | klotho beta | KLB | 3,2469 | -3,9559 | -12,8444 |
| NM_002594 | proprotein convertase subtilisin/kexin type 2 | PCSK2 | 26,7835 | 2,1851 | -12,2573 |
| NM_020318 NM_021936 | pappalysin 2 | PAPPA2 | 9,83817 | -1,20753 | -11,8799 |
| NM_002443 NM_138634 | microseminoprotein, beta- | MSMB | 9,9168 | -1,15399 | -11,4439 |
| NM_001828 | Charcot-Leyden crystal protein | CLC | 4,0715 | -2,57736 | -10,4937 |
| NM_000237 | lipoprotein lipase | LPL | -1,01815 | -10,3582 | -10,1735 |
| NM_003125 | small proline-rich protein 1B (cornifin) | SPRR1B | 60,4024 | 6,14865 | -9,82369 |
| NM_003225 | trefoil factor 1q | TFF1 | 51,4315 | 51,4315 | -8,79391 |
| NM_001461 | flavin containing monooxygenase 5 | FMO5 | 5,7606 | -1,52629 | -8,79236 |
| NM_001126102 ///  NM_005143 ///  NM_020995 | haptoglobin /// haptoglobin-related protein | HP /// HPR | 6,40461 | -1,34522 | -8,61562 |
| NM_014668 /// NM_033090 /// NM_148903 | GREB1 protein | GREB1 | 18,6357 | 2,16302 | -8,61562 |
| NM_032044 | regenerating islet-derived family, member 4 | REG4 | 9,21194 | 1,07808 | -8,54476 |
| NM_002769 | protease, serine, 1 (trypsin 1) | PRSS1 | 11,772 | 1,42133 | -8,28238 |
| NM_002630 | progastricsin (pepsinogen C) | PGC | 3,23631 | -2,95927 | -9,57711 |
